# Use of primary health care services and mortality in older patients with type 2 diabetes with or without comorbidities

**DOI:** 10.1080/02813432.2023.2255062

**Published:** 2023-09-14

**Authors:** E. Mellanen, T. Kauppila, H. Kautiainen, M. Lehto, O. Rahkonen, K. Pitkälä, M. K. Laine

**Affiliations:** a Department of General Practice and Primary Health Care, University of Helsinki and Helsinki University Hospital, Helsinki, Finland; b Folkhälsan Research Centre, Helsinki, Finland; c Primary Health Care Unit, Kuopio University Hospital, Kuopio, Finland; d City of Vantaa, Vantaa, Finland; e Department of Public Health, University of Helsinki, Helsinki, Finland

**Keywords:** Type 2 diabetes mellitus, mortality, service utilization, primary healthcare, comorbidity, aged

## Abstract

**Objective:**

This study aimed to examine primary health care (PHC) service utilization and mortality in older patients with type 2 diabetes (T2D) with or without comorbidities.

**Design and setting:**

A cohort study in PHC in the city of Vantaa, Finland. Follow-up period was set between the years 2011 and 2018.

**Subjects:**

PHC patients aged 60 years or more with a T2D were included.

**Main outcome measures:**

Service utilization was defined as the number of face-to-face appointments and telephone contacts between a patient and general practitioner (GP) or nurse. The presence of comorbidities was defined using the Charlson Comorbidity Index (CCI). Mortality was assessed using hazard ratio (HR) and standardized mortality ratio (SMR).

**Results:**

In total, 11,020 patients were included and followed for 71,596 person years. Mean age of the women and men in the beginning of follow-up were 71 and 69 years, respectively. The patients in the study cohort had a mean of eight appointments per person year to the GPs or nurses. Patients with T2D with comorbidities had more appointments than patients with T2D without comorbidities (incidence rate ratio (IRR) 1.44 [95% CI 1.39–1.49]). Increase in the number of all appointments reduced mortality in patients with T2D with and without comorbidities. Between patients with T2D with comorbidities and patients with T2D without comorbidities, the age and sex adjusted HR for death was 1.50 (95% CI 1.39–1.62). The SMR was higher in patients with T2D with comorbidities (1.83 [95% CI 1.74–1.92]) than in patients with T2D without comorbidities (0.91 [95% CI 0.86–0.96]).

**Conclusions:**

In older patients with T2D, the presence of comorbidities was associated with increased use of PHC services and increased mortality. Increase in the number of appointments was associated with reduced mortality in patients with T2D with or without comorbidities.

**Key Points**
- In older patients with T2D, it has not been studied whether and to what extend multimorbidity affects use of PHC services and mortality.
- The presence of comorbidities according to the Charlson Comorbidity Index (CCI) was associated with increased use of PHC services.
- The number of appointments to GPs or nurses was associated with reduced mortality in patients with T2D with or without comorbidities according to the CCI.

## Introduction

Type 2 diabetes (T2D) is a major cause of morbidity, and the health burden of T2D is increasing [1–3]. Diabetes is one of the 10 most important drivers of increasing burden, with a 24% age-standardized increase in disability-adjusted life year (DALY) rates [3]. The global prevalence of T2D is estimated to increase, although some European and East Asian countries have shown stable or decreasing trends in the incidence of T2D [1,4]. Furthermore, the burden of T2D in older people is estimated to almost double by 2030 [2].

Globally, most patients with T2D are treated in primary health care (PHC), and T2D is a common reason for PHC appointment [5–9]. In Finland, T2D is the fifth most common cause of general practitioner (GP) appointments [10]. Older T2D patients seem to use PHC services more often than age- and sex-matched controls without diabetes [11]. Furthermore, the use of PHC services in patients with T2D seems to increase with the duration of T2D [12,13]. In addition, the use of physicians, nurses and PHC services varies in T2D patients depending on the context, organization of health care and patient profile [14]. Multimorbidity is common in patients with T2D, and they have more comorbidities than those without diabetes [15].

A recent English cohort study in adult patients with T2D found an association between additional PHC appointments and increased rates of mortality [16]. However, the assessment of mortality lacked important factors such as the use of a reference population and standardized mortality ratio (SMR). The extent to which multimorbidity in older patients with T2D affects PHC utilization and mortality is also unknown.

This study aimed to examine the association between comorbidity and the use of PHC services and the association between mortality and the use of PHC services in patients with T2D aged 60 years or older.

## Methods

This cohort study was conducted in the city of Vantaa, Finland, which is located in the Helsinki metropolitan area. Vantaa is the fourth-most populated city in Finland [17]. In 2011, Vantaa had a population of 203,000 inhabitants of which 51% were women, 24% were 60 years or older and 11% had a foreign background [17]. Finland’s healthcare system is multifaceted. The health care system is mainly publicly funded, and the public sector is divided into PHC and specialized health care. In addition to public health care, there is a private sector and occupational health care sector [18]. Furthermore, in Finnish PHC, specially trained diabetes nurses, registered nurses and public health nurses are an active part in the treatment of patients with T2D to the extent where these nurses can even make changes to dosing of patients’ medications independently.

Due to changes in the organization of PHC services locally in the city of Vantaa, we set the inclusion period between 1 January 2002 and 31 August 2011, and the follow-up period between 1 September 2011 and 31 December 2018. During the inclusion and follow-up periods, the PHC of Vantaa city used an electronic health care system (Finnstar, Logica, Helsinki, Finland). This electronic health care system includes data on the age and sex of the patients, appointments to the GPs and nurses, diagnosis codes according to the International Classification of Diseases tenth revision (ICD-10), and prescription drug details with Anatomical Therapeutic Chemical (ATC) codes.

Patients were included in our study cohort if they were at least 60 years old at the beginning of the follow-up period and had a diagnosis of T2D made during the inclusion period. This age group was selected as the study cohort because patients in this age group mostly use public PHC services [19,20]. Patients were defined as having T2D if they had an ICD-10 code E11 or at least one prescription of an antihyperglycemic drug (ATC codes A10*) written by GPs. Data on sex, age at the beginning of the follow-up period, comorbidities relevant to the calculation of the Charlson Comorbidity Index (CCI), and the number of appointments to GPs or nurses during the follow-up period were gathered. Age at the beginning of follow-up, 1 September 2011, was used in all analyses. CCI was defined according to the CCI [21] using data from the inclusion period from 1 January 2002 to 31 August 2011. An exception in the calculation of CCI was made with diabetes, which was excluded from the calculation of CCI, and no age correction was performed. Patients were defined as having T2D with comorbidities if they had a CCI value of one or more, and T2D without comorbidities if they had a CCI value of zero. The comorbidity status acquired at the end of the inclusion period was used in all analyses. Patients with T2D with comorbidities and patients with T2D without comorbidities are later in this paper referred to as T2D-patients with comorbidities and T2D-patients without comorbidities, respectively. The appointments included in the analysis were face-to-face appointments and telephone contacts, and the number of appointments was reported per person year. Healthcare professionals whose appointments were included were GPs and nurses. Patients were followed up until the end of the follow-up period or until death, whichever occurred first. Data on the date of death were obtained from Statistics Finland (Helsinki, Finland). Mortality was assessed using hazard ratio (HR) and SMR.

Research permits for the data and this study were granted by the Research Ethics Committee of the Faculty of Medicine of the University of Helsinki (04/2019 and 06/2020), the city of Vantaa (VD/8059/13.00.00/2016 and VD/4713/13.00.00/2019), and Statistics Finland (TK-53-514-20).

Data are presented as means with standard deviations (SDs) or counts with percentages.

Crude and standardized estimates of care service incidence or incidence rate ratios (IRRs) were calculated using Poisson’s regression models. A possible nonlinear relationship between the number of appointments and the HR of death was assessed using 3-knot-restricted cubic spline regression. The length of the distribution of knots was located at the 25th, 50th and 75th percentiles. The knot locations were based on Harrell’s recommended percentiles [22]. Kaplan–Meier’s survival analysis were performed to estimate cumulative all-cause mortality. Adjusted Kaplan–Meier cumulative mortality rates were estimated using inverse probability weighting (IPW). Age and sex were introduced into the models as covariates when appropriate. The ratio between observed and expected numbers, the SMR, was calculated using subject-years methods with 95% confidence intervals (CIs), assuming a Poisson distribution. The expected number of deaths was calculated on the basis of sex-, age-, and calendar-period-specific mortality rates in the Vantaa population (Official Statistics of Finland, Helsinki, Finland). The expected number was determined by multiplying the person-years of observation by the appropriate mortality rate in the general population according to categories of sex, 1-year age group and calendar period. The Poisson regression was tested using the goodness-of-fit test of the model, and the assumptions of overdispersion in the Poisson model were tested using the Lagrange multiplier test. The proportional hazards assumption was tested graphically and using a statistical test based on the distribution of Schoenfeld residuals. All analyses were performed using STATA 17 .0 (StataCorp LP, College Station, TX).

## Results

Table 1 shows the characteristics of the study cohort. The mean ages of the women and men were 71 (SD 7.9) and 69 (SD 6.8) years, respectively. Of the T2D-patients included in the study cohort, 34% had comorbidities according to CCI. Among T2D-patients with comorbidities, the mean CCI was 2.9 (SD 1.3), and no difference was observed between men and women in the presence of comorbidities, with mean CCI values of 2.9 (SD 1.3) and 2.8 (SD 1.3), respectively.

**Table 1.** Characteristics and mortality of the patients aged 60 years or more with type 2 diabetes with or without comorbidities from the city of Vantaa, Finland.

	All patients	Patients with type 2 diabetes without comorbidities	Patients with type 2 diabetes with comorbidities	*p* Value
Number of persons	11,020	7267	3753	–
Women, *n* (%)	5503 (50)	3605 (50)	1898 (51)	.34
Age, years, mean (standard deviation)	70 (7)	69 (7)	72 (8)	<.001
Number of deaths, *n*	2682	1195	1487	
Person years followed up	71,596	49,103	22,493	–
Mortality^a^ (95% confidence interval)	24.3 (23.5–25.2)	16.4 (15.6–17.3)	39.6 (38.1–41.2)	<.001
Standardized mortality ratio (95% confidence interval)	1.26 (1.21–1.31)	0.91 (0.86–0.96)	1.83 (1.74–1.92)	<.001

Presence of comorbidities based on Charlson Comorbidity Index.

^a^ At the end of the follow-up period (Kaplan–Meier’s estimate).

**Figure 1.**
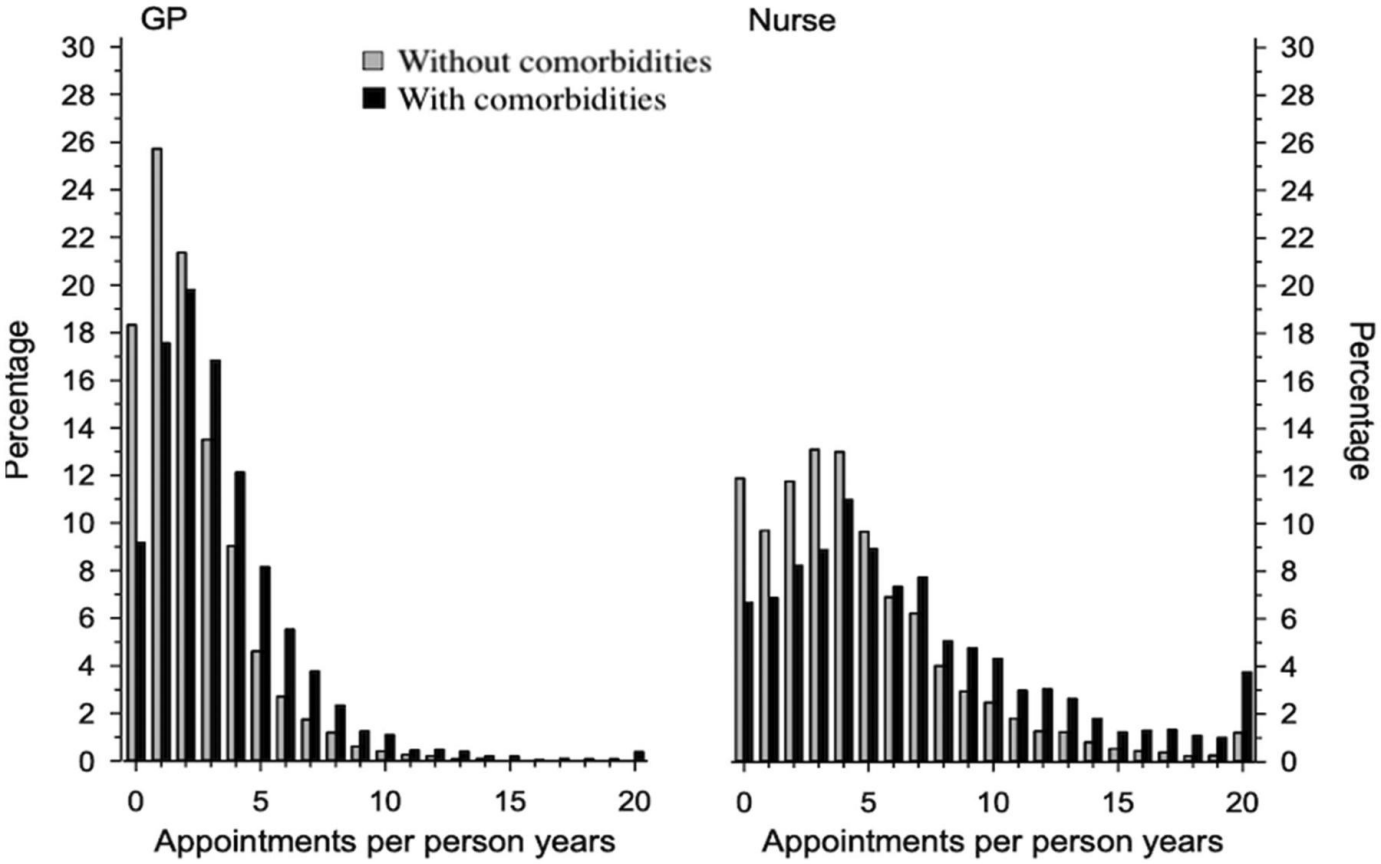
Distribution of general practitioner (GP) and nurse appointments per person years in the patients aged 60 years or more with type 2 diabetes with or without comorbidities from the city of Vantaa, Finland. Presence of comorbidities based on Charlson Comorbidity Index.

Figure 1 shows the distribution of appointments per person years to the GPs and nurses in T2D-patients with or without comorbidities. Altogether, the patients of the study cohort had a mean of 7.98 appointments per person years to the GPs or nurses. Table 2 shows the appointments per person years to the GPs and nurses. T2D-patients with comorbidities had more appointments to the GPs or nurses compared to T2D-patients without comorbidities (IRR 1.44 [95% CI 1.39–1.49]).

**Table 2.** General practitioner and nurse appointments per person years in patients aged 60 years or more with type 2 diabetes with or without comorbidities from the city of Vantaa, Finland.

	All patients with type 2 diabetes, mean (SE)	Patients with type 2 diabetes without comorbidities, mean (SE)	Patients with type 2 diabetes with comorbidities, mean (SE)	IRR^a^ (95% CI)
All appointments				
Women	8.46 (0.09)	7.54 (0.10)	10.42 (0.18)	1.38 (1.32–1.44)
Men	7.49 (0.09)	6.38 (0.10)	9.98 (0.19)	1.51 (1.43–1.58)
All	7.98 (0.07)	6.96 (0.07)	10.21 (0.13)	1.44 (1.39–1.49)
General practitioner appointments				
Women	2.80 (0.03)	2.51 (0.04)	3.41 (0.06)	1.33 (1.27–1.39)
Men	2.34 (0.03)	2.01 (0.03)	3.07 (0.06)	1.44 (1.37–1.51)
All	2.57 (0.02)	2.26 (0.02)	3.25 (0.04)	1.38 (1.33–1.43)
Nurse appointments				
Women	5.66 (0.07)	5.03 (0.07)	7.01 (0.14)	1.40 (1.34–1.47)
Men	5.15 (0.07)	4.37 (0.07)	6.91 (0.14)	1.54 (1.46–1.63)
All	5.41 (0.05)	4.7 (0.05)	6.96 (0.10)	1.47 (1.42–1.52)

IRR: incidence rate ratio; SE: standard error; CI: confidence interval.

Presence of comorbidities based on Charlson Comorbidity Index.

^a^ Between patients with type 2 diabetes without comorbidities and patients with type 2 diabetes with comorbidities, adjusted for age and sex.

**Figure 2.**
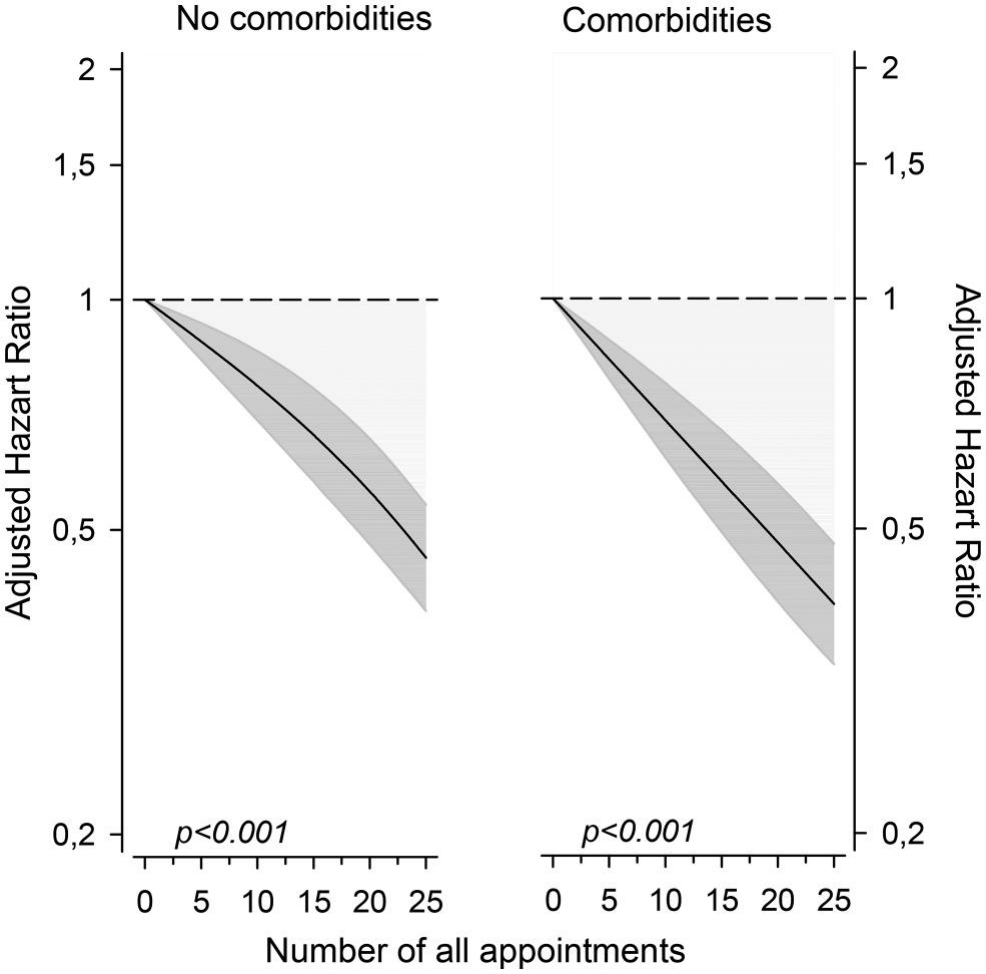
Association of the number of general practitioner and nurse appointments and mortality in patients aged 60 years or more with type 2 diabetes with or without comorbidities from the city of Vantaa, Finland. Presence of comorbidities based on Charlson Comorbidity Index. The number of appointments is expressed per person years and mortality is shown as age and sex adjusted hazard ratios.

The SMR was higher in T2D-patients with comorbidities than in T2D-patients without comorbidities (*p* < .001) (Table 1). Between T2D-patients with comorbidities and T2D-patients without comorbidities, the age and sex adjusted HR for death was 1.50 (95% CI 1.39–1.62). The number of GP and nurse appointments reduced mortality in both T2D-patients with comorbidities and in T2D-patients without comorbidities (Figure 2). In Figure 2, at the mean number of all appointments (eight appointments per person years), in T2D-patients without comorbidities and in T2D-patients with comorbidities, the HRs for death were 0.82 (95% CI 0.75–0.89) and 0.75 (0.68–0.82), respectively.

**Figure 3.**
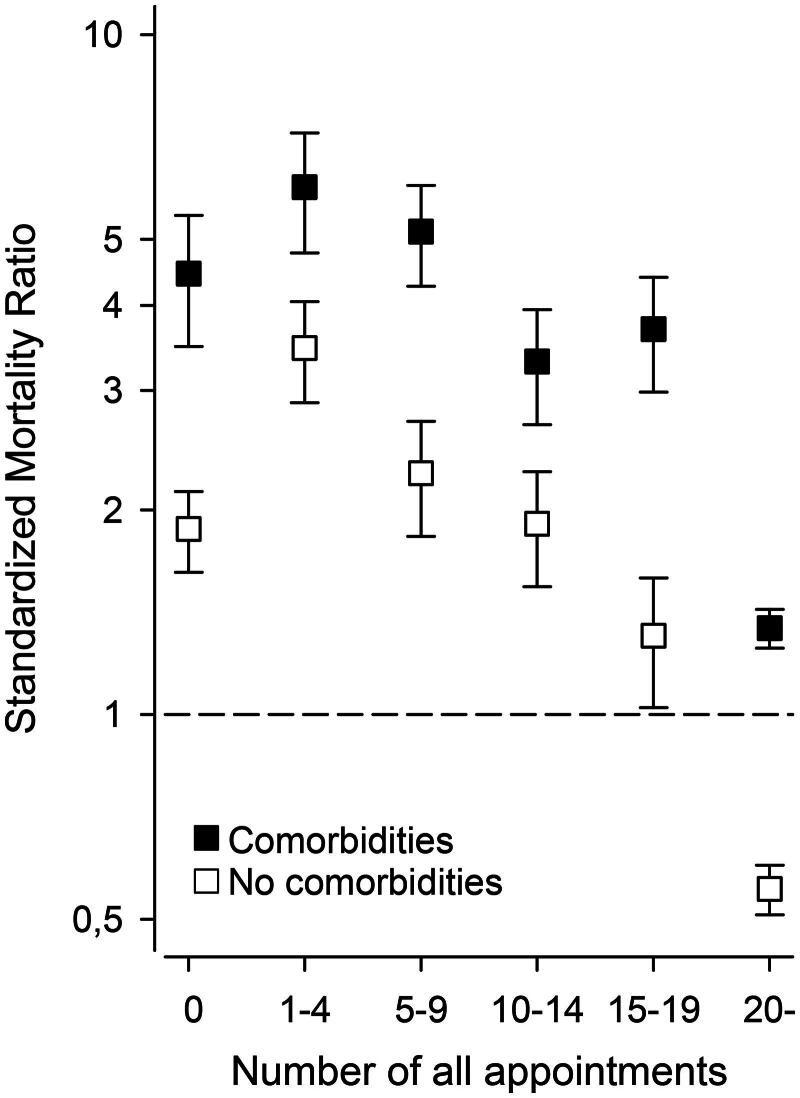
Comparison of standardized mortality ratio between population aged 60 years or more of the city of Vantaa, Finland, and patients with type 2 diabetes with or without comorbidities in different number of appointments per person years. Presence of comorbidities based on Charlson Comorbidity Index.

**Figure 4.**
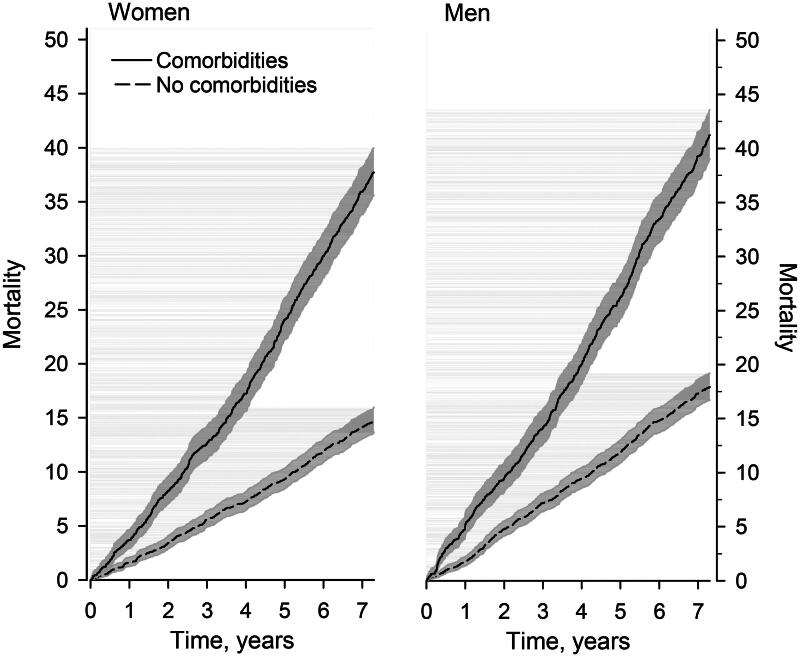
Age-adjusted Kaplan–Meier’s survival comparison in women and men with type 2 diabetes aged 60 years or more with or without comorbidities from the city of Vantaa, Finland. Presence of comorbidities based on Charlson Comorbidity Index.

In comparison with the population of Vantaa, T2D-patients with or without comorbidities had mostly higher SMRs (Figure 3) at different PHC service utilization rates. Figure 4 shows the age-adjusted Kaplan–Meier survival figures in women and men between T2D-patients with or without comorbidities. In women and men, the age-adjusted HRs for death between T2D-patients with comorbidities and T2D-patients without comorbidities were 2.08 (95% CI 1.86–2.33) and 2.07 (95% CI 1.86–2.30), respectively. A directed acyclic graph (DAG) between an individual exposure (use of PHC services) and outcome (mortality) is shown in Supplementary figure 1.

## Discussion

The present study found that T2D-patients with comorbidities had more GP and nurse appointments than T2D-patients without comorbidities. Furthermore, it was observed that the number of appointments to a GP or nurse reduced mortality in T2D-patients with or without comorbidities. Mortality in T2D-patients without comorbidities was lower than in T2D-patients with comorbidities.

This study has several strengths. The study cohort is a representative sample of patients with T2D aged 60 years or older because of the integrity of the electronic patient record system in a single city, a publicly funded health care system, and inclusion of only older patients. During the follow-up, the proportion of GP appointments with a documented diagnosis in the collected dataset was at a good level (approximately 90%), as shown in a previous study using the same dataset where PHC visits and documented diagnosis were examined [23]. Inclusion of all GP and nurse appointments and not focusing on only T2D related appointments gives a more complete view of the utilization of PHC services. The inclusion of nurse appointments is crucial in a nurse-driven T2D treatment system like ours in Finland to obtain an appropriate view of service utilization.

This study has some limitations. The organization and accessibility of healthcare services vary depending on country and context, and since the data of the study was obtained from a single city, the city of Vantaa, the generalizability of observed associations and presented appointment numbers is limited. The proportion of nurse appointments was high in this study and therefore the results presented may not be reproducible in health care system where appointments are concentrated to a GPs alone. The use of PHC services was examined, but the total use of all health care services remains unclear because the collected data did not include information from specialized health care, the private sector or occupational health care. In addition, continuity of care and its effect on mortality was not studied. Potential mediators of the results presented in this paper were not examined because the data of this study did not include laboratory tests or imaging study results. Further, the duration of comorbidities or T2D was unknown, and thus no further subanalyses were done.

This study found that PHC patients aged 60 years or more with T2D with comorbidities had an increased number of appointments to GPs or nurses. These findings are aligned with those of previous studies that have reported higher annual PHC service utilization rates in adult patients with T2D with comorbidities [16,24–26]. However, previous studies have varied in their study designs. All previous studies included patients with T2D aged 18 years or older, in contrast to this study’s older study cohort [16,24–26]. Various definitions of comorbidity or multimorbidity have been used. Two studies stratified patients with T2D according to their cardiovascular disease (CVD) status [24,26], one study used CCI [25], and one study defined comorbidities based on existing quality outcome framework (QOF) conditions, including coronary heart disease [16]. Furthermore, there are differences regarding which healthcare professional groups and which types of appointments are included in the studies. Two studies reported GP and nurse appointments [24,25] and two other studies reported the total number of contacts without specifying which healthcare professional contacts were considered [16,26]. One study reported face-to-face and telephone appointments, as was done in this study [25]. One study reported only in-person appointments [24], one study reported in-person appointments, telephone appointments and home visits [16] and one study reported outpatient visits, emergency room visits and inpatient hospital visits [26]. Despite differences in reported studies, T2D-patients with comorbidities tend to use more PHC services than T2D-patients without comorbidities.

The more appointments T2D-patients with or without comorbidities had to GPs or nurses, the lower was the patient mortality rate. Further, T2D-patients with comorbidities had higher mortality than T2D-patients without comorbidities. In addition, mortality in T2D-patients without comorbidities was lower in relation to the population of Vantaa. A previous English study by Lay-Flurrie et al. where no stratification by underlying diagnoses was done, found no association between PHC GP and nurse consultation rates and mortality [27]. Contrary to this study’s results, another English study by Hodgson et al. reported increased rates of mortality per additional PHC contact in patients with T2D [16]. In addition to the distortion caused by different healthcare systems between this study and the study by Hodgson et al., differences in study cohorts and determination of covariates might partially explain the different findings. This study’s study cohort was older, and even though Hodgson et al. used age adjustment in their analysis of mortality, the difference between the study cohorts age distribution is significant. Hodgson et al. included in-home visits, which were not included in this study. In this study, analysis was restricted to GP and nurse appointments only, whereas no specification of a health care professionals was done by Hodgson et al. Hodgson et al.’s study had a larger study cohort than this study, but as a strength of this study, in addition to HR, mortality was also examined using SMR. Use of SMR expands assessment of mortality to the level of population of the city this study was conducted in instead of only looking mortality inside a certain cohort. The lower SMR of T2D patients without comorbidities in relation to the population of Vantaa might be explained by the Finnish T2D treatment system, which is well resourced with specialized diabetes nurses focusing mostly on patients with diabetes, or factors not included in the data, such as lifestyle, smoking status or morbidity of the population of Vantaa. The frequency or number of PCH appointments in patients with T2D has been reported to improve glycemic control in patients with T2D who were formerly dropped out of the treatment system [28] and to reduce the occurrence rate of potentially avoidable hospitalizations [29]. These reported beneficial findings could provide some reasoning for the presented results showing reduced mortality with a higher number of PHC GP and nurse appointments in patients with T2D. Furthermore, the present study suggests topics for further research. Mortality seemed to increase in Figure 3 when annual PHC appointments increase from 0 to 1–4. If this increase in mortality with low annual PHC appointments compared with not visiting health centers at all is true, further studies would be needed to identify mechanisms involved (other comorbidities not involved in our current study, medicational aspects, issues related to organization of health services, patients’ behavioral mechanisms involved, etc.).

The present study observed that T2D-patients with comorbidities used more PHC services than T2D-patients without comorbidities. In a world of growing demands on healthcare systems, it would be beneficial to focus on preventative measures in patients with T2D to postpone the development of comorbidities and potentially reduce the use of PHC services. This study also demonstrated a reduction in mortality with a higher number of PHC appointments. Due to limited resources in healthcare, it is not meaningful or possible to pursue more frequent PHC appointments for every patient with T2D. Instead, it might be beneficial to target patients with T2D who do not use or use seldom PHC services in order to return them among services [28]. Future research is needed to identify factors and needs that shape patients’ use of PHC services, clarify the effect of PHC service utilization on mortality, and evaluate whether the assessment of continuity of care alters the results presented in this study.

In T2D-patients aged at least 60 years, the presence of comorbidities was associated with an increased use of PHC services measured as GP or nurse appointments. An increase in the number of GP or nurse appointments was associated with a reduction in mortality in both T2D-patients with or without comorbidities. A lower SMR in T2D-patients without comorbidities was observed in relation to the population of Vantaa.

## Supplementary Material

Supplemental Material

## References

[1] Khan MAB, Hashim MJ, King JK, et al. Epidemiology of type 2 diabetes – global burden of disease and forecasted trends. J Epidemiol Glob Health. 2019;10(1):107–111. doi: 10.2991/jegh.k.191028.001. PMC7310804 32175717

[2] Prince MJ, Wu F, Guo Y, et al. The burden of disease in older people and implications for health policy and practice. Lancet. 2015;385(9967):549–562. doi: 10.1016/S0140-6736(14)61347-7. 25468153

[3] GBD 2019 Diseases and Injuries Collaborators. Global burden of 369 diseases and injuries in 204 countries and territories, 1990-2019: a systematic analysis for the global burden of disease study 2019. Lancet. 2020;396(10258):1204–1222. 33069326 10.1016/S0140-6736(20)30925-9 PMC7567026

[4] Miao K, Wang Y, Cao W, et al. Trends in incidence of total or type 2 diabetes: systematic review. Twin Res Hum Genet. 2023;366:1–8.

[5] Bigio J, MacLean E, Vasquez NA, et al. Most common reasons for primary care visits in low- and middle-income countries: a systematic review. PLOS Glob Public Health. 2022;2(5):e0000196. doi: 10.1371/journal.pgph.0000196. 36962326 PMC10022248

[6] Wändell P, Carlsson AC, Wettermark B, et al. Most common diseases diagnosed in primary care in Stockholm, Sweden, in 2011. Fam Pract. 2013;30(5):506–513. doi: 10.1093/fampra/cmt033. 23825186

[7] Mash B, Fairall L, Adejayan O, et al. A morbidity survey of South African primary care. PLOS One. 2012;7(3):e32358. doi: 10.1371/journal.pone.0032358. 22442666 PMC3306367

[8] Soler JK, Okkes I, Oskam S, et al. An international comparative family medicine study of the transition project data from The Netherlands, Malta and Serbia. Is family medicine an international discipline? Comparing incidence and prevalence rates of reasons for encounter and diagnostic titles of episodes of care across populations. Fam Pract. 2012;29(3):283–298. doi: 10.1093/fampra/cmr098. 22308182

[9] Davidson JA. The increasing role of primary care physicians in caring for patients with type 2 diabetes mellitus. Mayo Clin Proc. 2010;85(12):S3–S4. doi: 10.4065/mcp.2010.0466. PMC2996164 21106869

[10] Hauhio N, Puroharju T, Mölläri K. Perusterveyden-huollon avosairaanhoidon vastaanoton asiakkaiden käyntisyyt vuonna 2020 [Reasons for visits by clients of primary health care outpatient clinics in 2020]. Finnish Institute for Health and Welfare; 2021. Available from: https://urn.fi/URN:NBN:fi-fe2021042820667

[11] Aro AK, Karjalainen M, Tiihonen M, et al. Use of primary health care services among older patients with and without diabetes. BMC Prim Care. 2022;23(1):233. doi: 10.1186/s12875-022-01844-2. 36085026 PMC9463776

[12] Weng W, Liang Y, Kimball ES, et al. Longitudinal changes in medical services and related costs in a single cohort of patients newly diagnosed with type 2 diabetes, 2006 to 2012. Clin Ther. 2016;38(6):1314–1326. doi: 10.1016/j.clinthera.2016.03.032. 27129399

[13] Sabale U, Bodegård J, Sundström J, et al. Healthcare utilization and costs following newly diagnosed type-2 diabetes in Sweden: a follow-up of 38,956 patients in a clinical practice setting. Prim Care Diabetes. 2015;9(5):330–337. doi: 10.1016/j.pcd.2015.01.001. 25631469

[14] van Dijk CE, Hoekstra T, Verheij RA, et al. Type II diabetes patients in primary care: profiles of healthcare utilization obtained from observational data. BMC Health Serv Res. 2013;13(1):7. doi: 10.1186/1472-6963-13-7. 23289605 PMC3570342

[15] Zghebi SS, Steinke DT, Rutter MK, et al. Eleven-year multimorbidity burden among 637 255 people with and without type 2 diabetes: a population-based study using primary care and linked hospitalisation data. BMJ Open. 2020;10(7):e033866. doi: 10.1136/bmjopen-2019-033866. PMC7358107 32611677

[16] Hodgson S, Morgan-Harrisskitt J, Hounkpatin H, et al. Primary care service utilisation and outcomes in type 2 diabetes: a longitudinal cohort analysis. BMJ Open. 2022;12(1):e054654. doi: 10.1136/bmjopen-2021-054654. PMC8808402 35105641

[17] Official Statistics of Finland (OSF): population structure [e-publication] [Internet]. Statistics Finland; 2023 [cited 2023 Feb 13]. Available from: http://www.stat.fi/til/vaerak/index.html

[18] Keskimaki I, Tynkkynen LK, Reissell E, et al. Finland: health system review. Health Syst Transit. 2019;21(2):1–166. 31596240

[19] Krakau I. Trends in use of health care services in Swedish primary care district. A ten year perspective. Scand J Prim Health Care. 1992;10(1):66–71. doi: 10.3109/02813439209014038. 1589667

[20] Hannikainen K. Ikääntyneiden sosiaali- ja terveyspalveluiden tarve ja käyttö eroavat tulotason mukaan [The need and use of social and health services in the elderly differs according to income level]. Finnish Institute for Health and Welfare; 2018. Available from: https://urn.fi/URN:ISBN:978-952-343-067-9

[21] Charlson ME, Pompei P, Ales KL, et al. A new method of classifying prognostic comorbidity in longitudinal studies: development and validation. J Chronic Dis. 1987;40(5):373–383. [Database] doi: 10.1016/0021-9681(87)90171-8. 3558716

[22] Fe H. Regression modeling strategies: with applications to linear models, logistic regression, and survival analysis. New York (NY): Springer; 2001.

[23] Lehto M, Mustonen K, Raina M, et al. Differences between recorded diagnoses of patients of an emergency department and office-hours primary care doctors: a register-based study in a Finnish town. Int J Circumpolar Health. 2021;80(1):1935593. 34077332 10.1080/22423982.2021.1935593 PMC8174484

[24] Abner S, Gillies CL, Shabnam S, et al. Consultation rates in people with type 2 diabetes with and without vascular complications: a retrospective analysis of 141,328 adults in England. Cardiovasc Diabetol. 2022;21(1):8. doi: 10.1186/s12933-021-01435-y. 35012531 PMC8744247

[25] Coles B, Zaccardi F, Seidu S, et al. Rates and estimated cost of primary care consultations in people diagnosed with type 2 diabetes and comorbidities: a retrospective analysis of 8.9 million consultations. Diabetes Obes Metab. 2021;23(6):1301–1310. doi: 10.1111/dom.14340. 33539642

[26] Mehta S, Ghosh S, Sander S, et al. Differences in all-cause health care utilization and costs in a type 2 diabetes mellitus population with and without a history of cardiovascular disease. J Manag Care Spec Pharm. 2018;24(3):280–290. 29485954 10.18553/jmcp.2018.24.3.280 PMC10397852

[27] Lay-Flurrie S, Mathieu E, Bankhead C, et al. Patient consultation rate and clinical and NHS outcomes: a cross-sectional analysis of English Primary Care Data from 2.7 million patients in 238 practices. BMC Health Serv Res. 2019;19(1):219. doi: 10.1186/s12913-019-4036-y. 30954074 PMC6451312

[28] Kauppila T, Eriksson JG, Honkasalo M, et al. Relationship between number of contacts between previous dropouts with type 2 diabetes and health care professionals on glycaemic control: a cohort study in public primary health care. Prim Care Diabetes. 2019;13(5):468–473. doi: 10.1016/j.pcd.2019.03.003. 30928432

[29] Georgescu V, Green A, Jensen PB, et al. Primary care visits can reduce the risk of potentially avoidable hospitalizations among persons with diabetes in France. Eur J Public Health. 2020;30(6):1056–1061. doi: 10.1093/eurpub/ckaa137. 32851398

