# Supplementary material for: Use of primary health care services and mortality in older patients with type 2 diabetes with or without comorbidities
Source: Scand J Prim Health Care. 2023 Sep 14;41(4):392–9. doi: 10.1080/02813432.2023.2255062 (PMC11001330; doi:10.1080/02813432.2023.2255062)
Supplement: Supplemental Material [file IPRI_A_2255062_SM5714.docx]

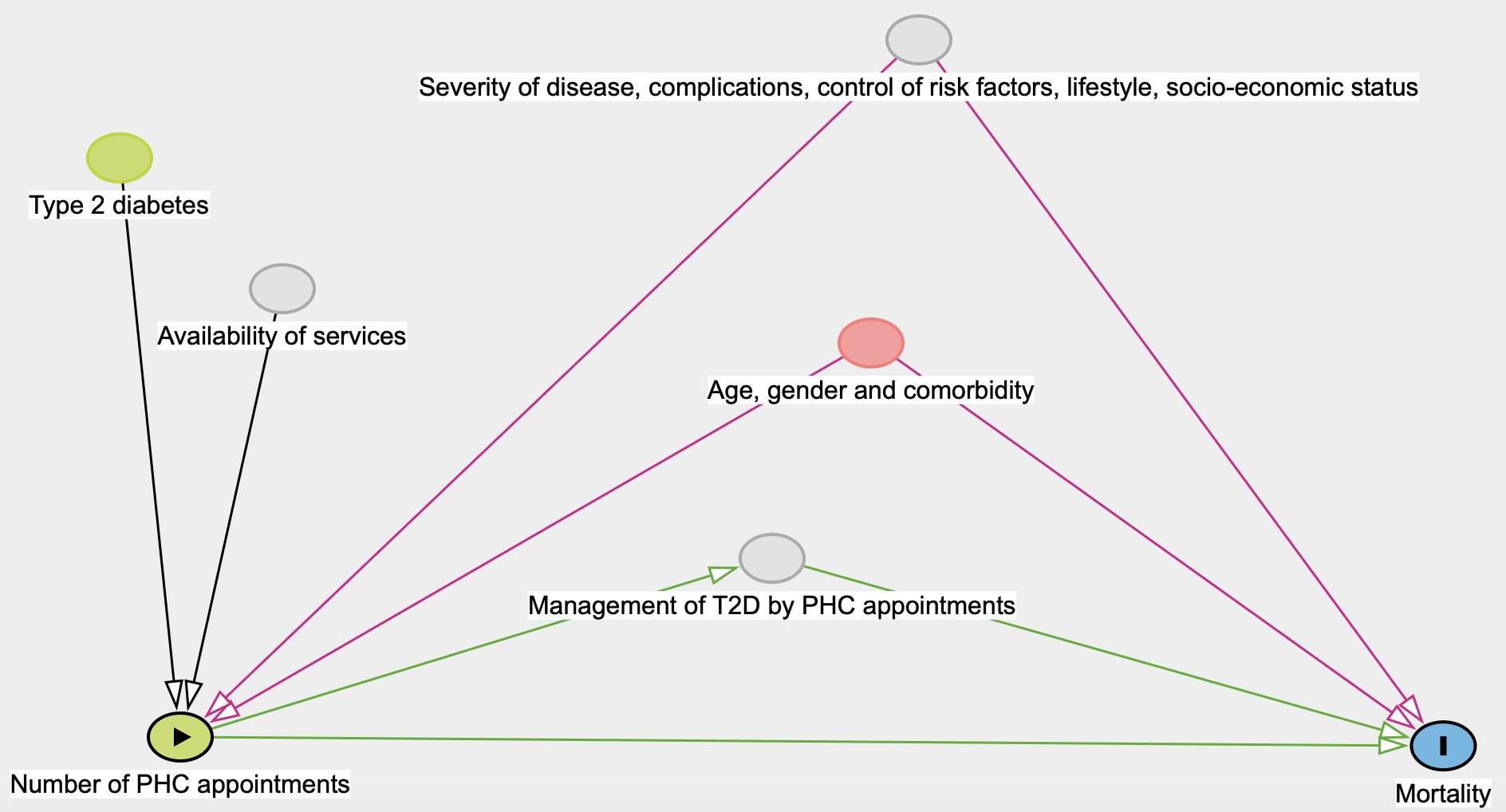


Supplementary figure 1 – Directed acyclic graph (DAG) illustrating studied exposure (Number of primary health care [PHC] appointments), outcome (Mortality) and associated factors with arrows representing direction of assumed causality. Grey circles represent factors that were not measured nor accounted for in analysis and red circle represents factors that were measured and used in analysis. Image created using DAGitty.net online tool.
